# Multi-Scale Deep Neural Network Based on Dilated Convolution for Spacecraft Image Segmentation

**DOI:** 10.3390/s22114222

**Published:** 2022-06-01

**Authors:** Yuan Liu, Ming Zhu, Jing Wang, Xiangji Guo, Yifan Yang, Jiarong Wang

**Affiliations:** 1 Changchun Institute of Optics, Fine Mechanics and Physics, Chinese Academy of Sciences, Changchun 130033, China; liuyuan18@mails.ucas.ac.cn (Y.L.); wangjing@ciomp.ac.cn (J.W.); guoxiangji18@mails.ucas.ac.cn (X.G.); yangyifan17@mails.ucas.ac.cn (Y.Y.); wangjiarong@cust.edu.cn (J.W.); 2 School of Optoelectronics, University of Chinese Academy of Sciences, Beijing 100049, China

**Keywords:** semantic segmentation, deep learning, dilated convolution, multi-scale, DeepLabv3+

## Abstract

In recent years, image segmentation techniques based on deep learning have achieved many applications in remote sensing, medical, and autonomous driving fields. In space exploration, the segmentation of spacecraft objects by monocular images can support space station on-orbit assembly tasks and space target position and attitude estimation tasks, which has essential research value and broad application prospects. However, there is no segmentation network designed for spacecraft targets. This paper proposes an end-to-end spacecraft image segmentation network using the semantic segmentation network DeepLabv3+ as the basic framework. We develop a multi-scale neural network based on sparse convolution. First, the feature extraction capability is improved by the dilated convolutional network. Second, we introduce the channel attention mechanism into the network to recalibrate the feature responses. Finally, we design a parallel atrous spatial pyramid pooling (ASPP) structure that enhances the contextual information of the network. To verify the effectiveness of the method, we built a spacecraft segmentation dataset on which we conduct experiments on the segmentation algorithm. The experimental results show that the encoder+ attention+ decoder structure proposed in this paper, which focuses on high-level and low-level features, can obtain clear and complete masks of spacecraft targets with high segmentation accuracy. Compared with DeepLabv3+, our method is a significant improvement. We also conduct an ablation study to research the effectiveness of our network framework.

## 1. Introduction

Since the beginning of the 21st century, human beings have accelerated the pace of space exploration, and space technology plays a vital role in it. Rapidly evolving computer vision and machine learning techniques have facilitated space technology development through applications in tasks such as collision avoidance self-navigation systems, spacecraft health monitoring, and asteroid classification [1,2]. The on-orbit formation and operation of large space equipment, such as space stations and the work and maintenance of spacecraft, cannot be completed without the support of space technology. Performing space missions often requires spacecraft images acquired by vision sensors as inputs, using computer vision technology to determine spacecraft position and attitude information, and then performing complex tasks, such as spacecraft rendezvous and docking, space equipment assembly on-orbit, spacecraft grasping and maintenance, and space debris removal [3,4,5]. In recent years, many spacecraft have been launched into space, the number of space exploration missions has increased, and the low Earth orbit has become crowded. Defunct satellites and space debris are growing and urgently need to be cleaned up [6]. Spacecraft positioning and other related issues have attracted more and more attention from researchers in space technology. The successful segmentation of spacecraft objects in the image is the key to the accurate positioning of spacecraft. The fine and precise spacecraft mask obtained after segmentation is conducive to the keypoint detection of the object, which is crucial for the realization of vision-based attitude estimation [7]. This paper investigates this issue.

Spacecraft image segmentation is to separate the spacecraft object and background from the acquired image. Compared with object detection, spacecraft image segmentation can obtain more accurate position information of the object and obtain more precise object contour, which is more difficult to achieve. Considering the particularity of the environment of the on-orbit mission, the spacecraft image segmentation task needs to face more severe lighting conditions, such as low light, overexposure, and spacecraft reflection, than the general image segmentation task. The impact of complex background and noise on the mission also needs to be considered. All these problems put forward higher requirements for the robustness and accuracy of the segmentation algorithm. Spacecraft objects can be divided into cooperative and non-cooperative spacecraft in different types of missions [8]. Cooperative spacecraft may be positioned and docked using dedicated radio links, reference markers, backward reflectors, etc. Non-cooperative spacecraft may be unknown spacecraft that do not have cooperative conditions. Cooperative spacecraft are designed with cooperative targets for precise docking at the grasping position for the on-orbit assembly mission. However, before docking is performed, it is necessary to operate the manipulator appropriately to ensure that the cooperative marks of the spacecraft are within the field of view of the hand-eye camera. Using spacecraft image segmentation techniques, even if the target spacecraft is non-cooperative, the spacecraft position can be obtained as long as the appearance of the spacecraft is known. The European Space Agency (ESA) and Stanford University held a competition in 2019 to use supervised learning to estimate the position and attitude of a known spacecraft from an image [9]. This paper belongs to this research direction but only studies the positioning of known spacecraft through monocular images.

In the past decade, deep learning has developed rapidly. Image segmentation tasks have achieved far better results than traditional machine learning methods through deep neural networks. Several branches, such as semantic segmentation, instance segmentation, and panoptic segmentation, have been developed. Image segmentation for known spacecraft is well suited for processing using deep learning methods. Researchers can build a spacecraft dataset with labelled information based on specific spacecraft application scenarios and train the network until the model converges. During inference, the network can quickly make predictions on the images and derive spacecraft segmentation results. However, space data are sensitive, and it is difficult to establish a real space object dataset. Some publicly available segmentation datasets, such as the Pascal Visual Object Classes (Pascal VOC), the Microsoft Common Objects in COntext (COCO), and the Cityscapes, are unsuitable for validating spacecraft segmentation algorithms [10,11,12]. The publicly available spacecraft datasets, such as Spacecraft Pose Estimation Dataset (SPEED) and the Unreal Rendered Spacecraft on Orbit (URSO), are mainly used to solve the attitude problem and lack the annotation of segmentation information [4,9]. Therefore, based on SPEED and URSO, we selected many photorealistic spacecraft images for refined annotation and constructed a spacecraft image segmentation dataset. We do not use the same annotation method as the spacecraft component segmentation dataset established in Ref. [7]. Our research is more concerned with whether the entire spacecraft can be accurately segmented. At the same time, we distinguish different kinds of spacecraft in the dataset annotation. We use a semantic segmentation network to solve the problem of spacecraft image segmentation. We also design the internal structure model of a deep neural network.

**Figure 1:**
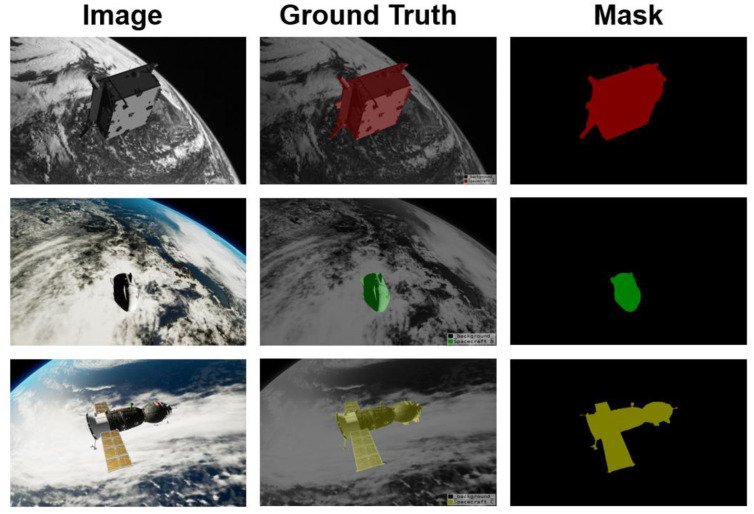
The display of our work.

Inspired by DeepLabv3+ [13] and DenseASPP [14], we propose a dilated convolution-based multi-scale neural network for spacecraft image segmentation, which we call SISnet. The network uses DeepLabv3+ as the basic framework and adopts the encoding and decoding form proposed by U-Net [15]. We use the spacecraft image segmentation dataset we made as the experimental dataset to verify the segmentation ability of the network model. Specifically, in the backbone network part, our network model uses dilated residual networks (DRN) [16] as the encoder for deep feature extraction. Our encoder utilizes dilated convolution, enabling the network to increase the receptive field while maintaining image resolution. Compared with residual networks [17], our backbone network removes the residual structure and skip-connections in the last few layers, which improves the gridding artifacts. Inspired by FarSee-Net [18] and Bai et al. [19], we utilize two atrous space pyramid pooling (ASPP) modules in the network to form a parallel-ASPP structure. The high-level and low-level features extracted from the backbone network are fused in the parallel-ASPP structure to improve the segmentation effect of the model for multi-scale targets. In addition, inspired by SENet [20] and ECA-Net [21], we have added a Squeeze-and-Excitation Module (SEM) between the backbone network and the parallel-ASPP structure. SEM is a channel attention mechanism module, which enables the network to pay more attention to some feature maps by giving channel weights. The innovation of this paper is that, through dilated convolutional and the parallel-ASPP structure, our network enhances contextual information and mitigates the effects of gridding artifacts. At the same time, the channel attention mechanism is used to recalibrate the features, which improves the learning ability of the network. Our method is more robust to noise in the image and can segment more complete and clear spacecraft masks. We experimented with SISnet on the spacecraft image segmentation dataset we produced above and compared it with other deep learning neural network methods. We experimented with SISnet on a spacecraft image segmentation dataset we produced and compared it with other deep learning neural network approaches. Experiments show that our method achieves a higher level with better segmentation results. Our work and predicted mask effects are shown in Figure 1.

The contributions of our paper are summarized as follows:

1. We designed an end-to-end segmentation network for spacecraft objects and produced a spacecraft image segmentation dataset to validate the algorithm.
2. We optimized the backbone network and used dilated convolution to increase the receptive field while maintaining the image resolution. With only 53 layers, our backbone achieves better results than ResNet-101 with more layers and Xception-65 with a larger number of parameters.
3. We added the channel attention mechanism to the segmentation network of the encoder–decoder structure to form an encoder + attention + decoder structure. Our network focuses on both high-level and low-level feature branches to improve the learning effect of the segmentation network.
4. We designed a parallel-ASPP structure. Using the superposition of different dilated convolutions, our network achieves multi-scale feature fusion with different-depth feature maps, enabling the network to segment the contours of spacecraft objects at different scales more clearly and completely.

The rest of the paper is organized as follows: in Section 2, we present the related work. In Section 3, we detail our proposed method. In Section 4, we detail the network training, comparison experiments, and ablation study of our approach. Quantitative and qualitative analyses are performed for the core benefits and performance of our network. In Section 5, we discuss the drawbacks of our method and the problems encountered during the research. Finally, in Section 6, we conclude the article.

## 2. Related Work

### 2.1. Image-Based Space On-Orbit Service Technology

In recent years, on-orbit service concepts, such as space debris removal, satellite acquisition, and spacecraft construction, have attracted more and more attention from academia and industry [9]. Space technology, such as spacecraft object detection and on-orbit service based on image processing, has gradually contributed to the research field of space object monitoring. The image processing technology is rapidly driving the progress of space technology. Sun et al. proposed an algorithm for adaptive real-time detection of faint optoelectronic geosynchronous orbit (GSO) debris [22] to detect a large amount of space debris in GSO that threatens the safety of spacecraft [23,24]. Through image adaptive fast registration and dilated difference algorithms, combined with mathematical morphology, threshold segmentation, and global nearest neighbor (GNN) multi-target tracking algorithms, Sun et al. achieved image background suppression, alignment, suspected target extraction, and multi-object tracking. This method can detect dim geostationary Earth orbit (GEO) and non-GEO debris in GSO. Khan et al. designed an image-based visual servo system [25]. They used the visual servo system for the first time to successfully track a super-orbital re-entry of a spacecraft and record its spectrum feature. They optimized the visual servo system by adding a simplified feedforward control dynamic model, and they verified the tracking performance of the system on the International Space Station (ISS).

Sharma et al. started research on monocular vision-guided on-orbit service technology in 2015 [26]. According to the explicit on-orbit service mission requirements of the United States and other countries [24,27], to approach space objects in an energy-efficient, safe, and accurate way, they began to explore relying on three-dimensional computer models and a single two-dimensional image for the initial pose estimation of space objects. They wanted to estimate the initial pose without using reference marks, without using any distance measurements or any prior relative motion information. Since then, the research in space object monitoring has also started to evolve from the detection and tracking of space objects to the precise acquisition of object position and attitude. This is very consistent with the direction of rigid body 6D pose estimation, which deep learning has rapidly developed in recent years. Almost all the pose estimation methods need to separate the object from the complex background, so most methods are segmentation-driven pose estimation. The precise mask of the object is crucial for subsequent processing, such as keypoint detection and pose discriminator [28].

In 2019, Stanford University and the ESA hosted the Satellite Pose Estimation Challenge, limiting input data to two-dimensional images and moving away from object 3D model data. This competition is very suitable for the application scenario where the object is a non-cooperative spacecraft without a target and the competition is also very challenging. Kisantal et al. proposed that the pose estimation of spacecraft could be divided into positioning and attitude determination, and the position error and attitude error were compared, respectively [9]. The classic monocular image-based space object positioning method iteratively solves the object position by extracting the shape context of the target, such as Harris corners, Canny edges, Hough transform, SIFT, SURF, and ORB features [29,30,31,32,33,34]. The Weak Gradient Elimination (WGE) technique was introduced by Sharma et al. [35]. It uses simple geometric constraints to synthesize the detected features to separate the edge features of the spacecraft from the weak edge features of the background. This method greatly reduces the search space of the feature correspondence problem. A more effective approach in recent years has been the use of deep neural network methods. It implements feature extraction through convolutional neural networks and uses datasets for supervised training, allowing the algorithm to predict the position of spacecraft quickly. In 2019, Sharma et al. [36] used a convolutional neural network combined with an advanced object detection algorithm to detect the 2D boundary box of the spacecraft in the image. Pedro et al. [4] adopted the ResNet [17] architecture with pre-trained weights as the network backbone. They removed unnecessary pooling layers to preserve spatial feature resolution to compress CNN features. In 2021, Dung et al. [7] built the first dataset for space object detection and segmentation, which annotated different components of the spacecraft. After training the dataset using deep learning methods, they could implement the components recognition. Dung et al. conducted experiments with multiple state-of-the-art detection and segmentation networks. They benchmarked the dataset, but they lacked improvements to the network structure and included no analysis of the experimental results. Our study improves the network structure and optimizes the effect of our model through structures such as channel attention mechanism and dilated convolution, and we have further analyzed and discussed the spacecraft segmentation issue.

### 2.2. Semantic Segmentation Based on Deep Learning

Since the advent of AlexNet [37], deep neural networks have begun to dominate image classification tasks and have shown excellent classification performance. FCN [38] adapted the classification network to a fully convolutional network and made significant progress by applying the network to semantic segmentation by classifying pixels. In order to enhance contextual information aggregation to improve the effect of semantic segmentation, some variants of FCN-based models have been proposed. SegNet [39] adopts an encoder–decoder structure that utilizes low-level information to help refine segmentation masks. U-Net [15] concatenates the outputs of low-level layers with those of high-level layers for information fusion. DeepLab [40] and CRF-RNN [41] use the conditional random field as post-processing for structure prediction in scene parsing. DPN [42] implements semantic segmentation using Markov random fields. PSPNet [43] builds a pyramid structure and fuses middle-level and high-level semantic features to obtain multi-scale context information.

In semantic segmentation tasks, contextual information plays an important role in image understanding to improve segmentation quality. Dilated convolutions can increase the receptive field without losing information by inserting cavities of different rates into regular convolutions. Deeplabv2 [44] and Deeplabv3 [45] embed contextual information using an ASPP, which uses parallel dilated convolution with different rates to fuse multi-scale information and expand the receptive field. DeepLabv3+ [13] adds a decoder structure while using the Xception with depthwise separable convolution as the backbone. It refines the segmentation results and makes the boundary of the object segmented more clearly. Visin et al. [46] proposed using recurrent neural networks to retrieve correlations in the global space.

In recent years, attention mechanisms have begun to be applied to deep learning tasks. From machine translation to image classification, attention mechanisms have demonstrated excellent performance. The self-attention mechanism [47] has been pioneered to achieve good results in machine translation by extracting the global dependencies of the input. SENet [20] proposes a lightweight channel attention mechanism that adaptively recalibrates the channel feature maps by establishing interdependencies between channels through the “Squeeze-and-Excitation” (SE) block. BAM [48] and CBAM [49] design attention mechanisms for both channel and spatial dimensions in a similar way to achieve adaptive feature refinement. PSANet [50] makes the network segmentation mask more sensitive to position and category information by the point-wise spatial attention module to adaptively aggregate contextual information for each point. DANet [51] expanded the self-attention mechanism in the segmentation task. It utilizes two attention mechanisms, which combine adaptive local features with their global dependencies to capture rich contextual relations. SKNet [52] designed a selective kernel unit that fuses the effective receptive fields of neurons of different sizes through branches of different kernel sizes. EMANet [53] proposed to describe the attention mechanism as an expectation-maximization method using the expectation-maximization attention module to perform mask estimation iteratively. Table 1 below shows a comparison of our proposed approach with several related methods. The first line indicates: whether to use the segmentation method, whether to adopt a deep learning strategy, whether the network design is carried out according to the characteristics of the spacecraft object, and whether relevant datasets have been established.

## 3. Methods

The SISnet network proposed in this paper is an end-to-end self-supervised learning network. The network adopts the structure of encoder+ attention+ decoder, which can effectively improve the segmentation accuracy of spacecraft objects. The network uses dilated convolution encoder to extract deep features. Through the channel attention mechanism, the network calibrates the feature information and innovatively pays attention to the calibration of the low-level features branch. Then, SISnet fuses features through a parallel-ASPP structure to enhance contextual information. Finally, the decoder of the network decodes the fused feature maps. The monocular images are input to the SISnet, which outputs spacecraft mask images.

### 3.1. Overall Network Architecture

**Figure 2:**
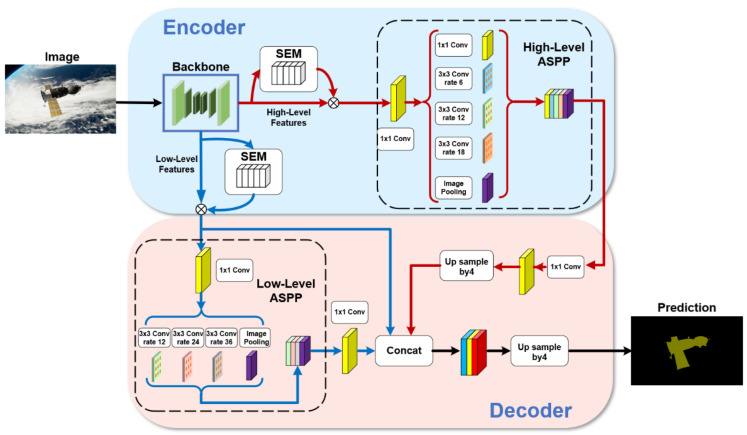
The structure of the semantic segmentation network for spacecraft images.

Our network is improved with DeepLabv3+ [13] as the baseline. Although DeepLabv3+ has achieved outstanding results in image segmentation, there are still some problems in the spacecraft image segmentation task, such as blurred object boundary segmentation, incomplete contour, inaccurate segmentation pixels, and image noise affecting the learning effect of the model. In order to solve these problems of DeepLabv3+, we have carried out a series of optimizations. First, in the backbone, we did not choose Xception-65 or ResNet-101, used by DeepLabv3+ for feature extraction. We use the DRN-D-54 [16] as a feature extraction network and achieve better results than Xception-65 or ResNet-101, with only about the same number of layers as ResNet-50. After the deep feature extraction in the backbone, the extracted features are divided into two different branches: high-level features and low-level features. Then, both branches should enter the SEM module of the channel attention mechanism to calibrate the channel feature response adaptively to improve the representation quality generated by the network. Finally, we improve the decoder structure. We add the second ASPP module. The two ASPP modules form a parallel ASPP structure. The high-level features enter the first ASPP module, and the low-level features enter the second ASPP module. In addition, the network still retains the low-level features branch without passing through ASPP module, which is fused together with the high-level and the low-level features passed through the ASPP module in the decoder structure. After a 3 × 3 convolution, the fused features are up-sampled by four times bilinear interpolation, and, finally, the predicted spacecraft mask is obtained. We will describe the details of the improvements in the following sections. The overall network structure is shown in Figure 2.

### 3.2. Network Backbone

In the backbone, we use an architecture DRN composed of dilated convolution and residual network as the encoder for feature extraction. The residual network achieves excellent performance on image classification tasks through structures such as multiple convolutional layer cascades and residual linking. The residual network achieves excellent performance on image classification tasks through structures such as numerous convolutional layer cascades and residual connections. Compared with the ordinary network, the residual network causes less information loss and a better learning effect and has become the preferred feature extraction tool for various neural networks. However, the residual network inevitably gradually reduces the resolution inside the convolutional network so that some spatial structures are no longer easy to distinguish. While image segmentation is a pixel-level image classification task, the loss of spatial acuity reduces the accuracy of image segmentation. DRN increases the receptive field of higher layers by setting dilated convolution to compensate for the reduction in the receptive field caused by replacing some down-sampling in the residual network. DRN enables the convolutional network to ensure higher resolution without changing the receptive field. Moreover, DRN removes some of the maximum pooling in the residual network that is no longer necessary and the residual connection at the back of the network. It improves the accuracy of image segmentation without changing the depth and complexity of the model.

**Figure 3:**
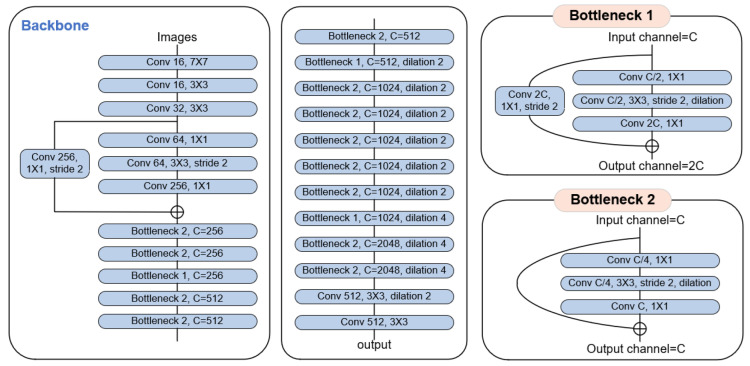
The structure of the network backbone. Each shape represented as a convolutional layer is actually a Conv-BN-ReLU group consisting of a convolutional layer, a BatchNorm layer, and a ReLU activation layer. The stride represents the step of the convolution. Dilation represents the rate adopted by dilated convolution. The letter C denotes the number of channels in the corresponding layer. 1 × 1, 3 × 3, and 7 × 7 are denoted as the kernel size of different convolution kernels.

There are several variants of DRN. Specifically, the structure we use is DRN-D-54. The structure diagram of the network is shown in Figure 3. We use a BatchNorm layer and a ReLU activation layer for each convolutional layer, forming a Conv-BN-ReLU group. DRN-D-54 is designed with three ordinary convolutional layers at the front of the network. In the middle of the network, similar to ResNet, DRN-D-54 has many bottleneck layers, some of which use dilated convolution. The bottleneck structure contains convolution with a kernel size of 1 × 1, which can change the network dimension more flexibly and reduce the computation of the network [17]. Depending on whether the number of channels varies, the DRN-D-54 design has two different bottleneck layers. When the number of channels in the bottleneck layer changes, additional convolutional layers are connected at the residual connection. At the back of the network, there is a dilated convolutional layer and a standard convolutional layer. The low-level features have only passed through 12 convolution layers, while the high-level features pass through all convolution layers in the DRN-D-54 network.

**Figure 4:**
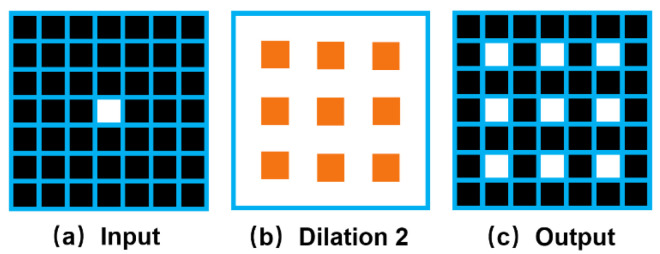
The gridding artifacts are caused by dilated convolution. (**a**) represents the input of a point pixel. (**b**) represents a layer of dilated convolution. (**c**) represents the output after dilated convolution. A single point pixel is mapped into multiple.

Dilated convolution improves the resolution of the output feature map without reducing the receptive field of individual convolution kernels, which has been proved to improve the segmentation performance in many segmentation networks. However, not all the convolutions can be replaced by dilated convolutions to achieve good results because dilated convolution may lead to gridding artifacts. As shown in Figure 4, assuming that the original image has only a point pixel, there are nine discrete pixel blocks in the feature map after dilated convolution. This gridding artifacts phenomenon will make the feature map relatively rough, showing a fine point-like distribution so that the segmentation results are not fine enough. Especially in the case of noise in the image, it will greatly affect the final segmentation result. Because there is a large amount of dilated convolution in Xception-65, there is noise in our data. In experiments, Xception-65 is not easier to converge, and it performs worse segmentation than many shallower networks or networks without dilated convolution. In order to remove the negative effects of these gridding artifacts, the degridding operation must be performed in the network. In ResNet, maxpooling at the front end of the network leads to output high-frequency high-amplitude activation values, and these high-amplitude activations are then easily propagated down by the later convolution and form the gridding artifacts after the dilated convolution. Therefore, DRN-D-54 removes it at the front end of the network. To avoid the residual connections that superimpose the gridding artifacts of the previous layer onto the next layer, the structure of DRN-D-54 last two layers is relatively simple. Compared to ResNet, we remove the residual connections from the last two layers and set a low dilation rate to help reduce the gridding artifacts. Therefore, the penultimate dilation rate is set smaller than the previous dilation rate [16]. Performing multiple dilated convolutions in succession may aggravate the gridding artifacts. Because the feature map after feature extraction of the backbone has to go into the attention and decoder structures, we do not use the dilation convolution in the last layer. These can make our backbone have a smoother feature map and affect our segmentation results. The experimental part verified that DRN-D-54 as a backbone achieved better segmentation results with fewer layers than Xception-65 and ResNet-101.

### 3.3. Squeeze-and-Excitation Attention Module

**Figure 5:**
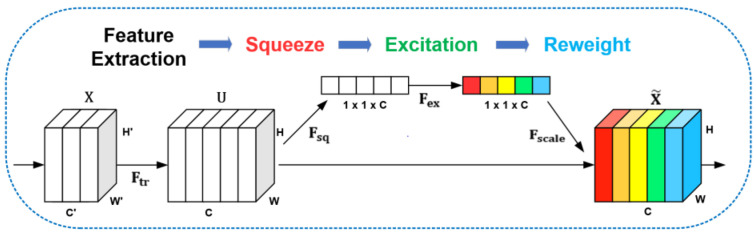
The schematic diagram of SEM.

To enhance the effect of feature extraction, we add an attention mechanism in the network. Inspired by SENet [20], we designed the Squeeze-and-Excitation Module (SEM) in the network. SEM uses channel attention to enable the network to obtain the importance of each feature channel by learning automatically. Its schematic diagram is shown in Figure 5. We calibrate the high-level and the low-level features through SEM and then enter the decoder structure at the back of the network. We verify that attention modulation on two branches of different depths has better results than focusing only on the high-level features.

SEM is a computing unit that can divide into three computing operations: Squeeze, Excitation, and Reweight, as shown in Figure 5. For a given image input $X\in {\mathbb{R}}^{H^{\prime }\times W^{\prime }\times C^{\prime }}$, the input is transformed into a feature map $U\in {\mathbb{R}}^{H\times W\times C}$ through the feature extraction process of $F_{tr}$. $F_{tr}$ can represent a convolution operation. $V=\left[v_{1},v_{2},\;\ldots ,v_{C}\right]$ is represented as the set of convolution kernels. $v_{C}$ represents the parameter of the *c*-th kernel. $x^{s}$ denotes the s-th output. $U=\left[u_{1},u_{2},\;\ldots ,u_{C}\right]$ as the output of this process. The transformation of the feature map can be expressed by the following formula:

(1) $$u_{c}=v_{c}*X=\overset{C^{\prime }}{\underset{s=1}{\sum }}v_{c}^{s}*x^{s}$$

Here, ∗ represents the convolution operation. $v_{c}=\left[v_{c}^{1},v_{c}^{2},\;\ldots ,v_{c}^{C^{\prime }}\right]$, $X=\left[x^{1},x^{2},\;\ldots ,x^{C^{\prime }}\right]$ and $u_{c}={\mathbb{R}}^{H\times W}$. $v_{c}^{s}$ represents a single channel in $v_{c}$, corresponding to the input X. After the feature map U is generated, the network officially enters the SEM. The first is the Squeeze operation. SEM uses global average pooling to compress the feature graph U along the spatial dimension H×W, turning each two-dimensional feature channel into a real number. In this operation, the input of H×W×C is transformed into the output $z\in {\mathbb{R}}^{C}$ of 1×1×C through the process of $F_{sq}$. z is the set of real numbers corresponding to channel number C. $z_{c}$ represents the *c*-th element of z, and its calculation formula is as follows:

(2) $$z_{c}=F_{sq}\left(u_{c}\right)=\frac{1}{H\times W}\overset{H}{\underset{i=1}{\sum }}\overset{W}{\underset{j=1}{\sum }}u_{c}\left(i,j\right)$$

This real number can be regarded as having a global receptive field, and the dimension of the output matches the number of feature channels of the input. It characterizes the global distribution of the response over the feature channels and makes the global receptive field available for the layers close to the input. Next is the excitation operation. SEM uses two fully connected layers to fuse the feature map information of each channel and then uses the ReLU function and the sigmoid function so that the network can learn the dependencies between channels through end-to-end training. That enables full capture of channel-wise dependencies. SEM takes the result z obtained by the Squeeze operation as input and transforms it into s through the process of $F_{ex}$. The formula is as follows:

(3) $$s=F_{ex}\left(z,W\right)=\sigma \left(g\left(z,W\right)\right)=\sigma \left(W_{2}\delta \left(W_{1}z\right)\right)$$

Here, $W_{1}\in {\mathbb{R}}^{\frac{C}{r}\times C}$ and $W_{2}\in {\mathbb{R}}^{C\times \frac{C}{r}}$ represent two fully connected layers. The dimension of z is 1×1×C and becomes $1\times 1\times \frac{C}{r}$ after the first full connection layer $W_{1}$. r denotes the scaling parameter of the fully connected layer, which can reduce the amount of computation. δ denotes the ReLU function and does not change the dimension of the output. After the second fully connected layer $W_{2}$, the output dimension becomes 1×1×C. Finally, the output s is obtained by the sigmoid function. s is the weight of feature maps in U, which is obtained by learning the fully connected layer and nonlinear layer. Finally, there is the reweight operation. The network considers the weight s of the excitation output as the importance of each feature channel after feature selection and then weights it to the previous feature map U by a channel-wise multiplication operation to obtain the output $\tilde{X}=\left[{\tilde{x}}_{1},{\tilde{x}}_{2},\;\ldots ,{\tilde{x}}_{C}\right]$. The weight completes the recalibration of the original features in the channel dimension. For the *c*-th ${\tilde{x}}_{c}$ element in $\tilde{X}$, SEM obtained the final ${\tilde{x}}_{c}$ by multiplying the two-dimensional matrix $u_{c}$ with each of the corresponding weights $s_{c}$ through the process of $F_{scale}$, as shown in the following equation.

(4) $${\tilde{x}}_{c}=F_{scale}\left(u_{c},s_{c}\right)=s_{c}u_{c}$$

**Figure 6:**
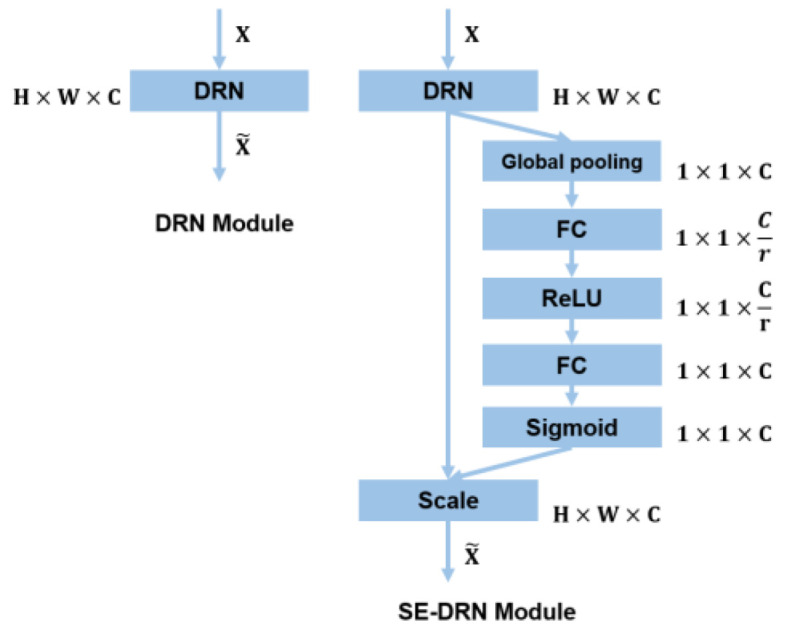
The dimensional change graph of the original DRN (**left**) and the SE-DRN module (**right**).

We added SEM between the DRN and the parallel-ASPP structure. In the Squeeze stage, the SEM module transforms the dimension of the input feature map from H×W×C to 1×1×C through global pooling. From the perspective of image resolution, both the high-level and the low-level features will be processed to the same size. Therefore, even with lower resolution feature maps, the SEM module can learn the channel-wise weights. In terms of module structure, SEM and DRN constitute the SE-DRN module, which guarantees the quality of feature extraction from the input image X to the output feature map $\tilde{X}$, as shown in Figure 6. The overall calculation of SEM is very small, and the input dimension is not changed. The channel attention mechanism enhances the useful feature channels, weakens the redundant feature channels, and can significantly improve the accuracy of noisy data. The SEM enables our network to have better adaptability to strong noise and highly redundant space data.

### 3.4. Parallel-ASPP

**Figure 7:**
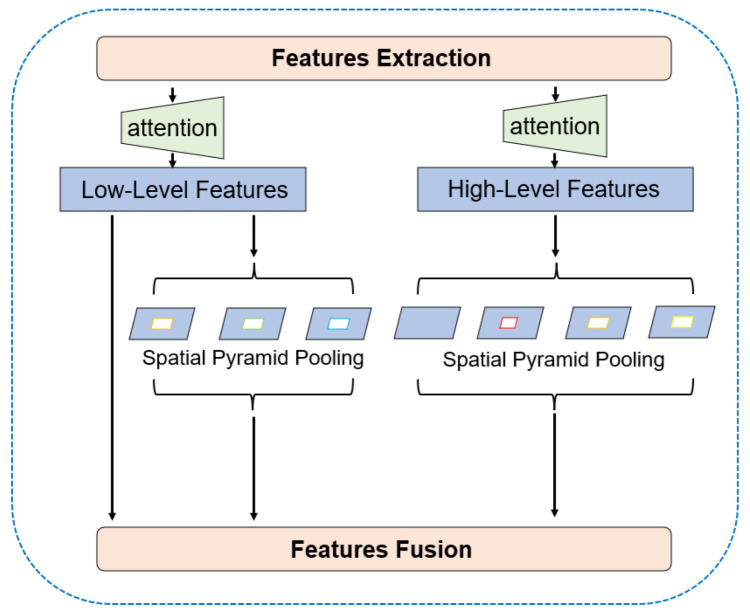
The schema of the parallel-ASPP structure.

To further capture and fuse image features at different scales, high-level and low-level features go into the parallel-ASPP structure we designed. The multiple dilation rates set by ASPP can help the network capture multi-scale contextual information, proven effective in both DeepLabv3 [45] and DeepLabv3+ [13]. However, DeepLabv3+ still leads to the problem of blurred segmentation boundaries. Inspired by Bai et al. [19], we added a second ASPP module to the decoder structure to further capture the multiscale contextual information of low-level features. Moreover, inspired by separable convolution [54] and Zhang et al. [18], we similarly change ASPP to factorized ASPP. The feature map goes through a separable convolution with a kernel size of 1 × 1, then into a parallel dilated convolution structure with different rates. This separated structure can be more convenient for us to adjust the number of channels of the network and can reduce the amount of calculation. In our SISnet, instead of duplicating the ASPP module, we put the high-level and low-level features into two different ASPP modules, as shown in Figure 7 below.

The ASPP module entered by the high-level features, after going through a 1 × 1 separate convolution, goes through a 1 × 1 standard convolution in parallel, three 3 × 3 dilated convolutions with dilation rates of [6,12,18], and a global average pooling layer. The module superimposes the parallel feature maps, then connects them by 1 × 1 convolution, and, finally, the feature maps are enlarged by four times bilinear interpolation up-sampling. In the ASPP module entered by the low-level features, the overall structure remains the same, and only changes are made in the parallel internal layers. It removes the 1 × 1 standard convolution and expands the dilation rate of the three-layer dilated convolution [12,24,36], which expands the receptive field of low-level features. Moreover, we retain a low-level features branch that does not enter the ASPP module and use the feature maps from SEM to perform subsequent feature fusion directly. This branch is equivalent to not entering two standard convolutional layers, reducing unnecessary computation. Finally, the feature maps of these three branches are combined for feature fusion. The fused features are fine-tuned by 3 × 3 convolution and then four times up-sampling using a bilinear interpolation method to predict the spacecraft mask. This asymmetric ASPP structure makes the segmentation boundary more complete and the semantic information clearer. These two ASPP modules form a parallel effect and fuse features together in the subsequent network, so we call it parallel-ASPP.

### 3.5. Loss Function

The loss function used in the SISnet network is the cross-entropy loss function. The formula is as follows:

(5) $$L=-\overset{N}{\underset{i=1}{\sum }}y_{i}log{\hat{y}}_{i}+\left(1-y_{i}\right)log\left(1-log{\hat{y}}_{i}\right)$$

Here, L is the training loss, N is the number of samples, $y_{i}$ is the real sample label, and ${\hat{y}}_{i}$ is the prediction label. The smaller the L value, the closer the prediction label is to the real sample label, and the more accurate the network segmentation result is.

## 4. Experiments

This section evaluates our proposed SISnet on the spacecraft segmentation dataset we labelled. In Section 4.1, we introduce the dataset and annotation details we used. In Section 4.2, we introduce the implementation details of our network and evaluation indicators. In Section 4.3, we compare our proposed method with representative methods in the segmentation domain: U-Net [15], HRNet [55], DeepLabv3+ [13], PSPNet [43], EMANet [53]. In Section 4.4, we conducted a comparative experiment on the backbone and a comparative experiment on the parallel-ASPP structure. In Section 4.5, we conducted an ablation study to demonstrate the effectiveness of our backbone, attention module, and decoder improvements.

### 4.1. Dataset

**Figure 8:**
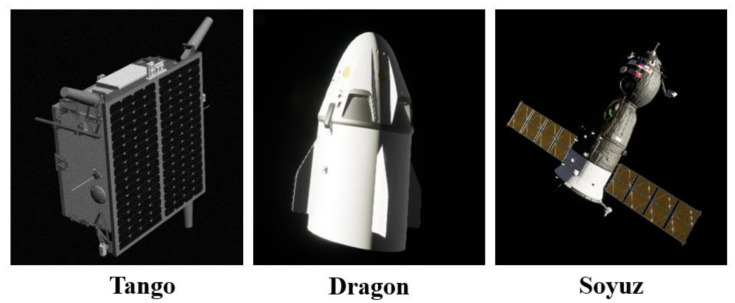
The appearance diagram of three types of spacecraft.

Currently, there are very few datasets that can be used for spacecraft image segmentation. We used the photorealistic spacecraft images provided in SPEED [9] and URSO [4], annotated by the LabelMe tool, and obtain fine masks of spacecraft targets. Based on this, we established a spacecraft segmentation dataset. The dataset contains three types of spacecraft, Tango, Dragon, and Soyuz. The appearance of each spacecraft is shown in Figure 8 below. All three spacecraft are morphologically different and representative. Choosing these three types of spacecraft as the dataset for training, rather than a single spacecraft, improves the variety of objects in our dataset and the difficulty of network learning while allowing us to demonstrate better generalization of our network.

**Figure 9:**
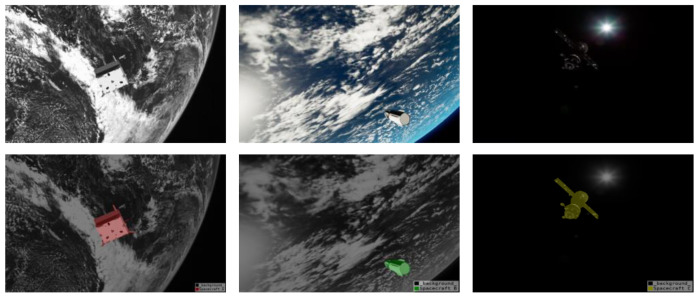
Diagram of the dataset. The first column is the original image, and the second column is the image displayed in an overlapping form after annotation.

During the labelling process, we marked different types of spacecraft with different colors. In addition, three objects are represented by the category names of Spacecraft A, Spacecraft B, and Spacecraft C. Their categories and color settings are shown in Table 2 below. The dataset consists of 600 monocular images with image sizes of 1280 × 960 and 1920 × 1200. Spacecraft A and Spacecraft B each have 150 images in the dataset, and Spacecraft C has 300 images. Since the morphology of Spacecraft C is more complex and often asymmetrical in the image, a larger amount of data were specially set for Spacecraft C. The effect of image annotation is shown in Figure 9 below. We divided the dataset into the training set, test set, and validation set in a 6:2:2 ratio. Finally, there are 360 training set images, 120 test set images, and 120 validation set images. The ratio of the three object categories in each subset is 1:1:2, which maintains the same data distribution. Among them, the training set and the validation set participate in the training process of the model. The validation set is used to avoid training overfitting and to determine the learning rate during training. We performed mask prediction and evaluation metrics on the test set to evaluate the training results of different models. Meanwhile, we used the resize operation to set the image size to 512 × 512 pixels in the network.

In remote sensing datasets, the size of the same type of object does not change much, so many deep learning models are anchor-based networks. The network can converge faster. However, even if it is the same object in the spacecraft picture, the size of the object may vary greatly due to different distances, so the spacecraft object has multi-scale characteristics. In addition, compared with the targets in the KITTI dataset, the spacecraft objects may appear in the image in any pose because of their motion characteristics. If the spacecraft structure is complex, then the changes reflected in the image may be more, which is the multi-attitude characteristics of the spacecraft data.

**Figure 10:**
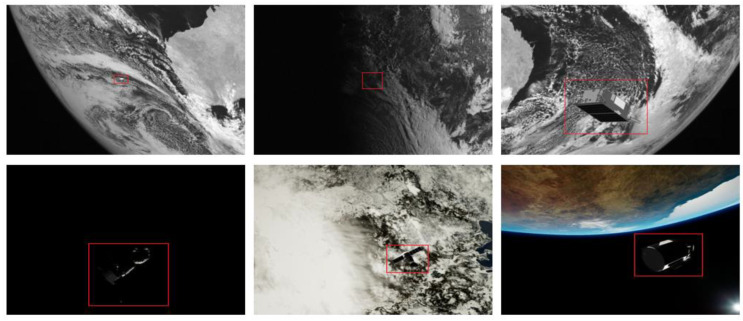
Diagram of hard examples in the dataset. Spacecraft objects are marked with red bounding boxes.

The background of the spacecraft is space, so the image will inevitably be affected by the illumination generated or reflected by various stars and spacecraft, resulting in large noise. Real data may even be much noisier than synthetic data. Spacecraft A is derived from the SPEED [9]. In order to improve the realism of the synthetic data, all images of Spacecraft A have been subjected to Gaussian blur processing (σ= 1) and zero mean, Gaussian white noise processing with ($\sigma^{2}=$ 0.0022). Due to the lighting conditions, there are often problems of underexposure or overexposure. The reflection on the surface of the spacecraft and the difficulty of identifying the structural parts of the spacecraft due to insufficient light will increase the difficulty of accurate target segmentation. Considering the influence of those mentioned above, multi-scale, multi-attitude, noise, and illumination imbalance, as well as the common complex backgrounds and weak targets in some public datasets, we selected the data when establishing the spacecraft segmentation dataset. Among them, there are 80 small-scale spacecraft images and 81 images of spacecraft in poor lighting conditions. There are 70 images of spacecraft with complex backgrounds. Our dataset contains more hard examples. The proportion of hard examples in the dataset reaches 38%. Some hard examples are shown in Figure 10 below. It can be seen that our spacecraft segmentation task is challenging and hard. Our dataset is a small-sample dataset. However, compared with public datasets commonly used for image segmentation, such as the Pascal VOC 2012, the number of images of our various objects is of the same magnitude. At this order of magnitude, our network can achieve segmentation well.

### 4.2. Implementation Details

**Figure 11:**
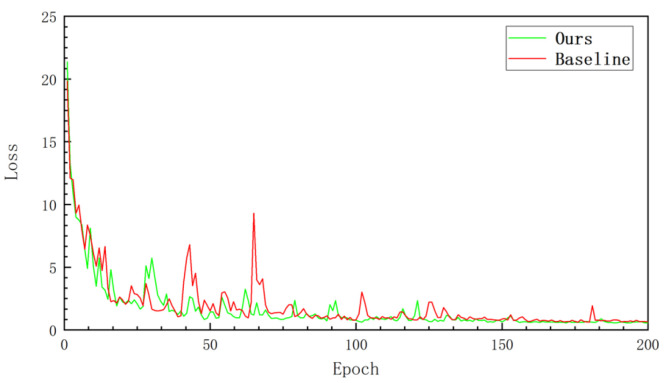
Network training loss curve.

The experimental environment is $\mathrm{Intel}\left(R\right){\mathrm{Core}}^{\mathrm{TM}}i9-9900K\;\mathrm{CPU}@3.60\mathrm{GHz}$, running memory 16 G, Ubuntu 18.04, 64-bit operating system. We use CUDA10.1(NVIDIA Corporation, Santa Clara, CA, USA), CuDNN 7.6.5 (NVIDIA Corporation, Santa Clara, CA, USA), python 3.7. We performed network training on an NVIDIA GEFORCE RTX2080Ti GPU. We trained our network with the open-source deep learning framework PyTorch. We used the Adam optimizer to update the weights of the neural network iteratively. The initial learning rate is set to 0.01 for network training. Other training parameters are shown in Table 3 below:

The SEM rate represents the scaling parameter of the SEM fully connected layer. The High-ASPP rate and Low-ASPP rate represent the dilation rate of parallel ASPP modules. The output stride represents the output stride of the encoder structure. We maintained consistent and comparable hardware and software parameters in each training and testing experiment. To focus on comparing network performance and exploring the impact of data on the network from experiments, we did not use data augmentation to expand our dataset further. Figure 11 shows the loss decline of our SISnet and our baseline method during training. It can be seen that, as the number of iterations increases, loss decreases rapidly. In the middle and later stages of training, our network loss fluctuates less and tends to stabilize more quickly.

### 4.3. Network Comparative Experiments

**Figure 12:**
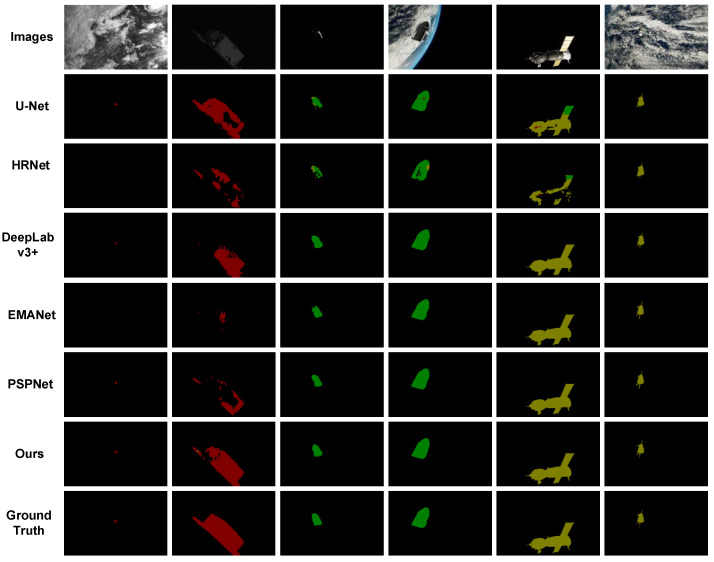
Experimental results and comparison with other methods.

Under the condition of the same training environment and training parameters, we conducted comparative experiments on various semantic segmentation networks. Figure 12 shows the prediction results of the network. From top to bottom are the original image, U-Net, HRNet, DeepLabv3+, EMANet, PSPNet, our proposed method, and the final truth image.

Overall, our network shows better segmentation results on these three types of objects, and the masks are smoother and fuller. To measure the segmentation effect of the network more accurately, we choose mean intersection over union (MIoU) as the evaluation metric and use the test set for quantitative analysis. When the value of MIoU is closer to 1, it indicates that the segmentation result is more accurate. The calculation formula of MIoU is as follows:

(6) $$\mathrm{MIoU}=\frac{1}{k-1}\overset{k}{\underset{i=0}{\sum }}\frac{p_{ii}}{\sum_{j=0}^{k}p_{ij}+\sum_{j=0}^{k}p_{ji}-p_{ii}}$$

In the above formula, k represents the number of categories, including the background, i represents the true value of the pixel category, and j represents the pixel prediction result. $p_{ii}$ represents the number of correctly classified pixels, and $p_{ij}$ represents the total number of pixels for which i is predicted to be j. $p_{ji}$ represents the total number of pixels for which j is predicted to be i. During training, we set up checkpoints to ensure that the highest MIoU weights are recorded. Meanwhile, our training MIoU can reach more than 94% after 200 epochs of training. We quantitatively evaluated the above networks on the test set, and the results are shown in Table 4 below.

It can be seen in Table 4 that EMANet uses ResNet-101 as the backbone and uses the expectation-maximization attention module to effectively improve the segmentation results. The combination of a deeper feature extraction network and the attention mechanism makes EMANet have better results on the test set for Spacecraft B and Spacecraft C. However, for Spacecraft A, which is noisy and has less color information, the result is not so good. Compared to U-Net and HRNet, our MIoU is higher because we have a deeper network. Compared with our baseline method, DeepLabv3+, we have greatly improved the IoU of all classes.

### 4.4. Structure Comparative Experiments

In this paper, we also experimented with the different backbone networks of the encoder. While keeping the baseline as DeepLabv3+, we replaced different backbones to compare the effect of different feature extraction networks in the spacecraft segmentation issue. We conducted experiments with Mobilenet-v2 [56], ResNet-50 [17], ResNet-101 [17], Xception-65 [13], Densenet121 [57], and DRN-D-54 [16]. The experimental results are shown in Table 5 below. Although Xception-65 performs well in some image segmentation tasks, its applicability to spacecraft objects is relatively poor due to noise and the gridding artifacts. Mobilenet-v2 is also a backbone using depthwise separation convolutions and achieved good results. ResNet-50 [D] and ResNet-101 [D] represent the dilated convolution versions of ResNet. ResNet-50 [D] and ResNet-101 [D] replace a small number of standard convolutional layers in the network with dilated convolutions with the dilation rate = 2 or 4. They increase the receptive field but do not cause serious gridding artifacts. ResNet-101 [D] achieved higher scores for the segmentation of Spacecraft A with a larger field of perception and deeper network depth. Meanwhile, because DRN-D-54 removes some residual connections, the focus on low-level features is not as good as ResNet-101 [D], so the segmentation effect of the network for Spacecraft A can be further improved when the decoder structure focuses on the low-level features. From the MIoU metric, ResNet-50 [D] and ResNet-101 [D] show that the network adds dilation convolution and demonstrate that dilation convolution is still a boost for the spacecraft segmentation task. To achieve higher segmentation results, we have to pay attention to maintaining the depth of the network and enhancing the contextual information while avoiding the gridding artifacts. Therefore, we adopted the encoder structure based on DRN-D-54. Experimental results show that our encoder has a good segmentation effect on all objects while achieving the highest MIoU.

Based on the above encoder experiments, while keeping the backbone network as DRN-D-54, we conducted experiments of adding a second ASPP module to the decoder structure. We let the low-level features in the baseline method directly enter the second ASPP module and then fuse with the high-level features passed through the ASPP module. The low-level features further aggregate contextual information through this process. During the experiments, we set different dilation rates for the second ASPP module. We achieved better segmentation results with fewer layers and larger dilation rates, as shown in Table 6 below. In the second experiment, we added a second ASPP structure that is identical to the first ASPP module. The segmentation results have been improved compared to the first experiment without adding the second ASPP module. However, in the third experiment, we removed the convolutional layer with the rate of 18, and the effect decreased instead because the low-level features require a larger receptive field to help improve the overall network effect. Therefore, we adjusted the convolutional layers in the parallel structure, as shown in the fourth experiment. We increased the dilation rate of convolution with all three layers only, which further improved the segmentation results. In the design of the final decoder, we also directly fused the low-level features through the channel attention with the features through the parallel ASPP structure to ensure the effect of attention modulation. We will show the experimental results in the ablation study.

### 4.5. Ablation Study

This section evaluates different network variables and analyses the reasons that affect network performance. We used DeepLabv3+ as the baseline, and the backbone of the baseline uses ResNet-101 with dilated convolution. We conducted improvement experiments with different network structures. (1) Baseline + A: indicates that the SEM attention module is embedded into the baseline method. (2) Baseline + B: indicates that DRN-D-54 replaces the backbone of the baseline method. (3) Baseline + D: indicates that our improved decoder structure is used. We first conducted experiments with single-variable improvement. Then, we conducted three groups: Baseline + A + B, Baseline + A + D, and Baseline + B + D. Finally, the experiment of our proposed SISnet method, namely Baseline + A + B + D, is conducted. We performed the quantitative evaluation on the test set for each experiment, and the experimental results are shown in Table 7 below.

As can be seen from Table 6, the segmentation results of our method compared to the baseline have been steadily improved. The DRN-D-54 backbone we used is optimized for the gridding artifacts and has a strong feature extraction ability. It can achieve better results than ResNet-101 with fewer layers. The channel attention mechanism we designed in the network can also improve the metric by about 1% by assigning channel weights. By enhancing the contextual information of low-level features, our decoder structure significantly improves the segmentation results of Spacecraft A, which is worth significantly improving the final MIoU. The ablation study validates the improvements we made. These improvements can improve the accuracy of spacecraft semantic segmentation to varying degrees. In terms of MIoU metrics, our approach achieves the best segmentation results.

## 5. Discussion

**Figure 13:**
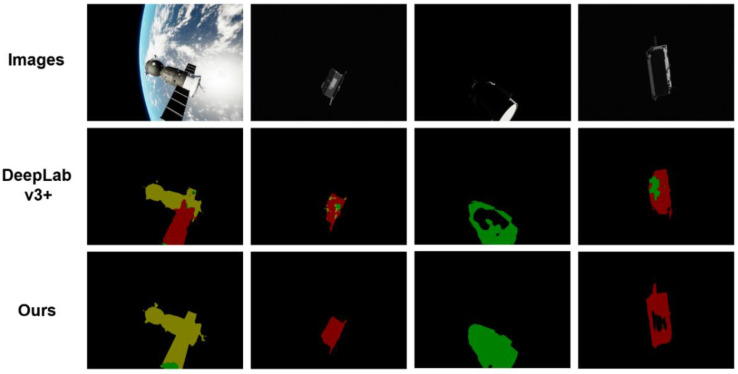
Failure cases.

We also found some problems in our research, briefly explained here. Due to the small test set of our dataset, if some image segmentation results are not good, it may cause relatively large changes in the MIoU value. Whether they are real data or realistic synthetic data, we must pay attention to the problem of noise in the network design because, in real tasks, noise is unavoidable. Our Spacecraft A is more regular in geometric structure and has salience in the image. The segmentation results of Spacecraft A are significantly improved when the effect of noise in the image is addressed. We show some failed cases in Figure 13 below. The second row in the figure is the segmentation result of the baseline method. The third row is the segmentation result of our method.

**Figure 14:**
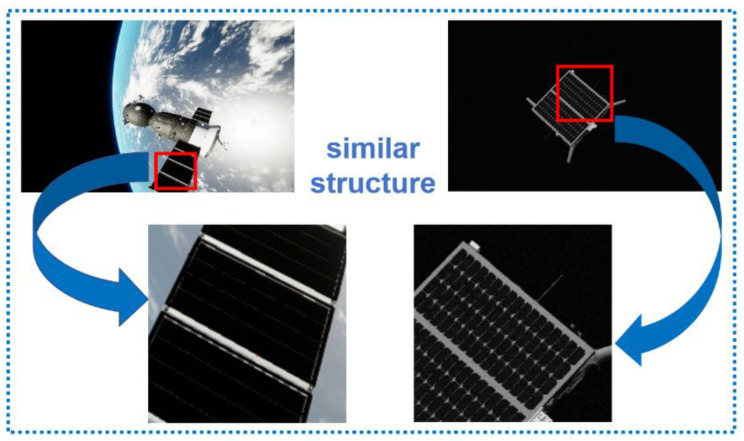
Comparison diagram of similar structures of different spacecraft.

The segmentation errors in the first column are caused by the similar structures of different types of spacecraft. As shown in Figure 14 below, the Spacecraft A and Spacecraft C objects have similar solar panel structures, and, in some perspectives of Spacecraft A, only the solar panels are more conspicuous. Similar structures are prone to the misclassification of pixels. There is also some degree of pixel clutter in the second column. It is related to the texture features similar to the spacecraft. Our network enhances the contextual information to alleviate such phenomena. The third and fourth columns of images are poor lighting conditions that make some structures invisible and make it easier for pixels to be misclassified as other spacecraft or space backgrounds. The segmentation results of DeepLabv3+ will have large cavities or misclassification. Our method can better segment a simply connected domain, but there are still cavity cases, and the segmentation effect can continue to improve in the future.

## 6. Conclusions

In this paper, an end-to-end spacecraft image segmentation network was proposed. Different types of spacecraft objects were segmented with monocular images as inputs to obtain their corresponding masks, and good segmentation results were achieved. We designed a neural network based on a dilated convolution and encoder+ attention+ decoder structure using the self-supervised learning method. Through the dilated convolutional and parallel-ASPP structure, our network enhanced the contextual information and mitigated the effects of gridding artifacts. In addition, the channel attention mechanism was introduced to recalibrate the features and improve the learning ability of the network. Our method is more robust to noise in the image and can segment complete and smooth object masks. We finely labelled public spacecraft datasets to establish a spacecraft segmentation dataset. We conducted various comparison experiments and an ablation study on the dataset. The experimental results show that our method outperforms other methods and has a better segmentation performance for spacecraft objects. In the future, we hope to improve the effect of spacecraft segmentation by the feature matching method in small-sample segmentation technology. At the same time, we would like to experiment with the deep unsupervised active learning strategy [58]. This allows the network to continuously acquire new knowledge for learning during the test phase. We also hope to build more complex space object segmentation datasets and evaluate our network more comprehensively.

Our work will contribute to the research on on-orbit assembly in space. It is helpful for the space manipulator to move quickly to the vicinity of the spacecraft through the image, which provides a basis for further target detection and docking missions.

## Figures and Tables

**Table 1:** The comparison table of related work.

Method	Segmentation	Deep Learning	Spacecraft Object	Dataset
Khan et al. [25]	no	no	yes	no
Sharma et al. [36]	no	yes	yes	Pose
Proenca et al. [4]	no	yes	no	Pose
Dung et al. [7]	yes	yes	no	Segmentation
DeepLabv3+ [13]	yes	yes	no	no
Ours	yes	yes	yes	Segmentation

**Table 2:** Classification number, name, and color table.

Number	Spacecraft	Category Name	RGB Value	Color
1	Tango	Spacecraft A	(128, 0, 0)	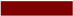
2	Dragon	Spacecraft B	(0, 128, 0)	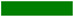
3	Soyuz	Spacecraft C	(128, 128, 0)	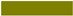
4	--	Background	(0, 0, 0)	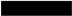

**Table 3:** Training parameters.

Parameter	Value	Parameter	Value
learning rate	0.01	SEM rate	16
epoch	200	batch size	2
High-ASPP rate	[1,6,12,18]	Low-ASPP rate	[12,24,36]
momentum	0.9	weight decay	0.0005
output stride	8	crop size	512 × 512

**Table 4:** The MIoU results of network comparative experiments on the test set.

Method	Spacecraft A	Spacecraft B	Spacecraft C	MIoU
U-Net [15]	64.42	83.51	90.13	79.35
HRNet [55]	40.04	32.59	56.9	43.18
DeepLabv3+ [13]	83.15	81.26	88.43	84.28
PSPNet [43]	83.61	81.88	93.84	86.44
EMANet [53]	74.38	89.12	94.18	85.89
Ours	93.11	87.62	92.87	91.20

**Table 5:** The MIoU results of backbone comparative experiments on the test set.

Backbone	Spacecraft A	Spacecraft B	Spacecraft C	MIoU
Mobilenet-v2 [56]	74.02	84.29	91.78	83.37
ResNet-50 [17]	81.01	77.1	90.09	82.74
ResNet-50 [D]	78.17	82.74	88.42	83.11
ResNet-101 [17]	77.64	82.71	88.7	83.02
ResNet-101 [D]	83.15	81.26	88.43	84.28
Xception-65 [13]	67.61	72.5	88.34	76.15
Densenet121 [57]	51.62	64.97	83.38	66.66
DRN-D-54 [16]	78.56	86.64	91.46	85.55

**Table 6:** The MIoU results of the second ASPP module comparative experiments on the test set.

Number	Low-ASPP Rate	Spacecraft A	Spacecraft B	Spacecraft C	MIoU
1	-	78.56	86.64	91.46	85.55
2	[1,6,12,18]	80.88	86.77	91.98	86.54
3	[1,6,12]	70.31	87.77	86.66	81.58
4	[12,24,36]	85.66	87.25	91.02	87.98

**Table 7:** The MIoU results of the ablation study on the test set.

Method	Spacecraft A	Spacecraft B	Spacecraft C	MIoU
Baseline	83.15	81.26	88.43	84.28
Baseline + A	84.87	82.74	88.11	85.24
Baseline + B	78.56	86.64	91.46	85.55
Baseline + D	91.87	83.83	90.61	88.77
Baseline + A + B	84.6	86.07	88.96	86.54
Baseline + A + D	93.25	84.15	91.5	89.63
Baseline + B + D	93.28	87.2	92.56	91.01
Baseline + A + B + D	93.11	87.62	92.87	91.20

## Data Availability

The SPEED dataset and the URSO dataset are made publicly available for research purposes. For more information, please refer to the websites https://kelvins.esa.int/satellite-pose-estimation-challenge/data/ (accessed on 4 May 2021) and https://zenodo.org/record/3279632 (accessed on 21 June 2021). For the data license, please refer to the websites https://creativecommons.org/licenses/by-nc-sa/3.0/legalcode (accessed on 20 May 2022) and https://creativecommons.org/licenses/by/4.0/legalcode (accessed on 20 May 2022).
